# A Susceptible Period of Photic Day-Night Rhythm Loss in Common Marmoset Social Behavior Development

**DOI:** 10.3389/fnbeh.2020.539411

**Published:** 2021-02-02

**Authors:** Mamiko Koshiba, Aya Watarai-Senoo, Genta Karino, Shimpei Ozawa, Yoshimasa Kamei, Yoshiko Honda, Ikuko Tanaka, Tohru Kodama, Setsuo Usui, Hironobu Tokuno

**Affiliations:** ^1^Engineering Department, Yamaguchi University, Ube City, Japan; ^2^Pediatrics, Saitama Medical University, Saitama, Japan; ^3^Graduate School of Information Sciences, Tohoku University, Sendai, Japan; ^4^Obstetrics and Gynecology, Saitama Medical University, Saitama, Japan; ^5^Tokyo Metropolitan Institute of Medical Science, Setagaya, Japan

**Keywords:** social developmental disorders, alert behavior, developmental age, adult expression, multivariate integration, principal component analysis, circadian rhythm

## Abstract

The prevalence of neurodevelopmental psychiatric disorders such as pervasive developmental disorders is rapidly increasing worldwide. Although these developmental disorders are known to be influenced by an individual’s genetic background, the potential biological responses to early life’s environmental exposure to both physical and psychological factors must also be considered. Many studies have acknowledged the influence of shorter time for rest at night and the simultaneous occurrence of various kinds of complications involving developmental disorders. In a prior study, we examined how a common marmoset’s (Callithrix jacchus) psychosocial development was affected when it was reared under constant daylight from birth and then reared individually by humans nursing them under constant light (LL) during their juvenile development stages. The behaviors of these marmosets were compared with those of normal day-night cycle (LD) marmosets using a multivariate analysis based on principal component analysis (PCA). That study found that LL marmosets relatively elicited egg-like calls (Ecall) and side-to-side shakes of the upper body with rapid head rotation through adulthood frequently. Based on the PCA, these behaviors were interpreted as “alert” or “hyperactive” states. However, we did not clarify susceptible periods of the photic rhythm loss experience and the psychological development output. In this study we summarize the following studies in our model animal colonies involving 30 animals (11 female, 19 males) to further explore critical age states of inquiry about each social behavior profiling. We compared social behaviors of three age stages, juvenile, adolescent and young adult equivalent to one another in four LL experience conditions, LL (postnatal day (P) 0 to around 150), Middle (P60–149, 90 days), Late (P150–239, 90 days), and LD (no experience). In the most representative 1st and 2nd principal component scores, the shifting to higher frequency of alert behaviors developed at the adult stage in LL, Middle, then Late in turn. The no LL experience group, LD, generally featured higher frequency of local preference of high position compared to LL experience present groups, in adulthood. This limited model primate study might inspire different developmental age sensitive mechanisms of neuronal network to control socio-emotional functions by utilizing the multivariate visualization method, BOUQUET. This study could potentially contribute to nurturing educational designs for social developmental disorders.

## Introduction

Throughout history, the number of hours humans remain active throughout a day has increased. The resulting social and environmental changes might have caused changes in the biological circadian mechanism of human beings (Baron and Reid, 2014; Mazzio and Soliman, 2014; Zitting et al., 2018; Moreno et al., 2019). A circadian rhythm abnormality is one of the major issues in developmental psychiatry reported in autism (Tordjman et al., 2015) and bipolar disorders (Gottlieb et al., 2019) characterized by social communication and emotional behavior impairments which needs further research on treatment design such as comprehensive intervention with environmental care (Arns et al., 2013), diet (Miller et al., 2015), home education (Raffington et al., 2018), and pharmacological treatment (Bourgeron, 2007). Among these considered factors, a photic day-night environmental rhythm applicable to humans and any other diurnal or nocturnal animals who form general circadian rhythms have been found pathogenesis, molecular, synaptic and neuronal mechanisms of cognition and behavior) using animal models in the field of developmental psychiatry (Homberg et al., 2016; Crossland et al., 2017; Sánchez-Vázquez et al., 2019).

We suggest a predictive method for visualizing psychological maldevelopment using a strategy of multivariate analysis called as BOUQUET [Behavior OUtput analysis for QUantitative Emotional state Translation (Senoo et al., 2011; Koshiba et al., 2013a,b,c,d, 2015, 2016; Mimura et al., 2013, 2015; Karino et al., 2020; Tao et al., 2020)] based on principle component analysis (PCA) of a subject’s behaviors. Since multiple kinds of epigenetic systems with susceptible period learning and the maldevelopment of neuronal circuit function have been reported (Klin et al., 2015; Hertz-Picciotto et al., 2018), further investigation of supports for psychological development and in relation neurodevelopmental. In the first report, we suggested that a non-human primate, a common marmoset, exhibiting maldevelopment be raised in a constant light environment without photic day-night rhythm (LL) against the two reference conditions of which one had a normal day-night cycle (LD) and another was reared under constant darkness (DD) (Senoo et al., 2011). The results revealed subtle differences between LL and LD groups as increased alert and hyper activity at the social context in adolescence. The report suggested that this marmoset LL model could be translated to human etiology with social interaction and communication deficits induced by irregular light rhythm. In a related report (Koshiba et al., 2015), we hypothesized that when 3–5 months old marmosets were exposed to constant light, their susceptibility to neurodevelopment impacts increased. Thus, this report explored the targeted age period and the earlier and later periods, this time to determine how constant light influences a common marmoset’s social behavior and development.

## Materials and Methods

### Animals

The experimental protocols were approved by the Animal Care and Use Committee of the Tokyo Metropolitan Institute for Neuroscience (2010–14). All the animal study experiment was operated in the Institute. Immediately after birth, 30 marmoset babies used in the study were isolated from their parents due to the parents’ inability to raise the babies by themselves. The babies were fed milk until weaning, normally around postnatal 3 months. They were housed individually in a light-sealed incubator illuminated by a fluorescent lamp maintaining constant temperature (32–28°C). After weaning, conventional chow and water were given *ad libitum* throughout the remainder of the experiment. Light intensity per cage was 750–930 lx during the light period, and 0 lx during the dark period. To examine the effects of photic environment during specific early life on behavioral development, the marmoset babies were raised under one of four lighting conditions LL (LL period; P0∼180d), LL- Middle (P60∼150d), LL-late (P150∼240d), LD (null), described in Table 1. To collect data on their social behavior for developmental trajectory analysis, we set three stages according to age (Senoo et al., 2011), (I) P134∼236, (II) P243∼320d, and (III) P338∼466. Experimental condition of both LL experience period and behavioral tests to be checked social character formation in each subject was shown in Figure 2F and the mean age with each standard deviation presented in Table 1. This experimental age duration was particularly designed LL-middle (P60∼150d) as a peri-weaning period and LL-late (P150∼240d) as a post-weaning period.

**TABLE 1:** Subject group conditions of LL.

LL, LD condition	Subject number f: female m: male	LL Period (postnatal day)	Behavioral Test Age average ± SD (postnatal day)		
			Period1 (P134∼236)	Period2 (P243∼320)	Period3 (P338∼466)
LL	f3, m4	0∼189	162.0 ± 28.0	−	419.3 ± 4.6
LL-early	f1, m4	60∼149	168.4 ± 5.3	262.8 ± 16.2	357.8 ± 22.8
LL-late	f1, m4	150∼239	187.5 ± 29.0	284.2 ± 28.0	427.0 ± 45.6
LD	f4, m7	−	190.1 ± 33.0	292.6 ± 15.1	382.1 ± 23.2

*Photic conditions abbreviate LL (constant light) and LD [12 h light (L) and 12 h dark (D)].*

### Behavior Output Analysis for Quantitative Emotional State Translation (BOUQUET)

Behavior Output Analysis for Quantitative Emotional State Translation (BOUQUET) was introduced and explained in our previous report (Senoo et al., 2011; Koshiba et al., 2015). BOUQUET is a strategy used to visualize complex behavioral factors to extrapolate psychological states quantitatively, a multivariate analysis based on principal component analysis (PCA) was followed by plotting the samples in the coordinates of representative components. Normally the first and second components are plotted with a time or age axis in a three dimension graph for use in linear and ellipse regression. We partially changed the measuring criteria to focus on socialization.

#### Social Behavior Test

**FIGURE 1:**
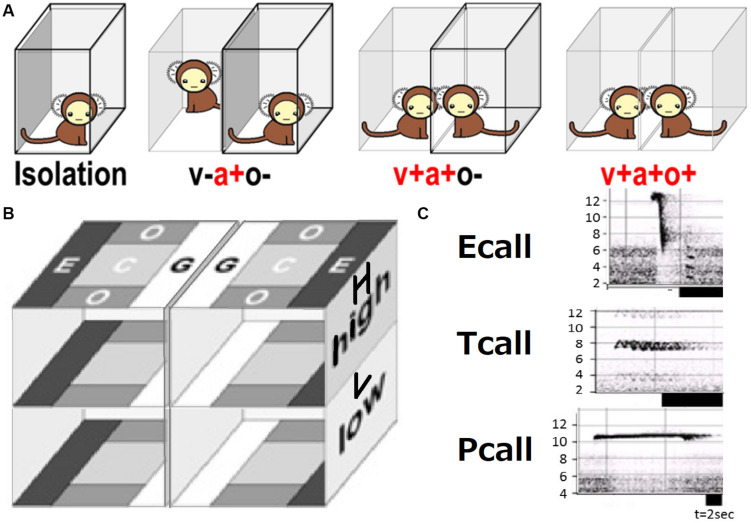
Social behavior test. **(A)** Four social contexts with (+) or without (–) sensory (v, visual; a, auditory; o, olfactory) interaction. **(B)** Local preference subarea terms (G, grouping; C, center; E, escape; O, other). **(C)** Typical spectrogram patters [frequency (kHz)] of three call types.

A series of contextual schemes of social behavior test is shown in Figure 1A. A subject in a transparent cage was exposed to another animal as follows in this order: (i) isolation (a-v-o-) covered fully with a transparent box, separated from the other cage by an opaque board with no conspecific present, (ii) plus acoustic cue (a + v-o-; with another animal present in another cage), (iii) plus visual (a + v + o-; the board was removed), (iv) plus olfactory (a + v + o +; the cover on the test cage was removed). To keep subjects as unfamiliar as possible, the possibility of meeting the same animal was limited during the test. All the subjects had no experience to meet the conspecific animals except once during the behavioral tests. We performed video-recording of an isolated subject [condition (i)] in the cage made of transparent, perforated vinyl chloride plates (bottom: 29 × 29 cm, height: ×45 cm), under fluorescent light at 350–600 lx inside the apparatus. Five digital video cameras (SONY, Japan) recorded the animals from the top and all four sides. Subjects were allowed to move freely within the cage. Vocal orientation assessment required five different microphones set to compare each sound wavelength and frequency.

**FIGURE 2:**
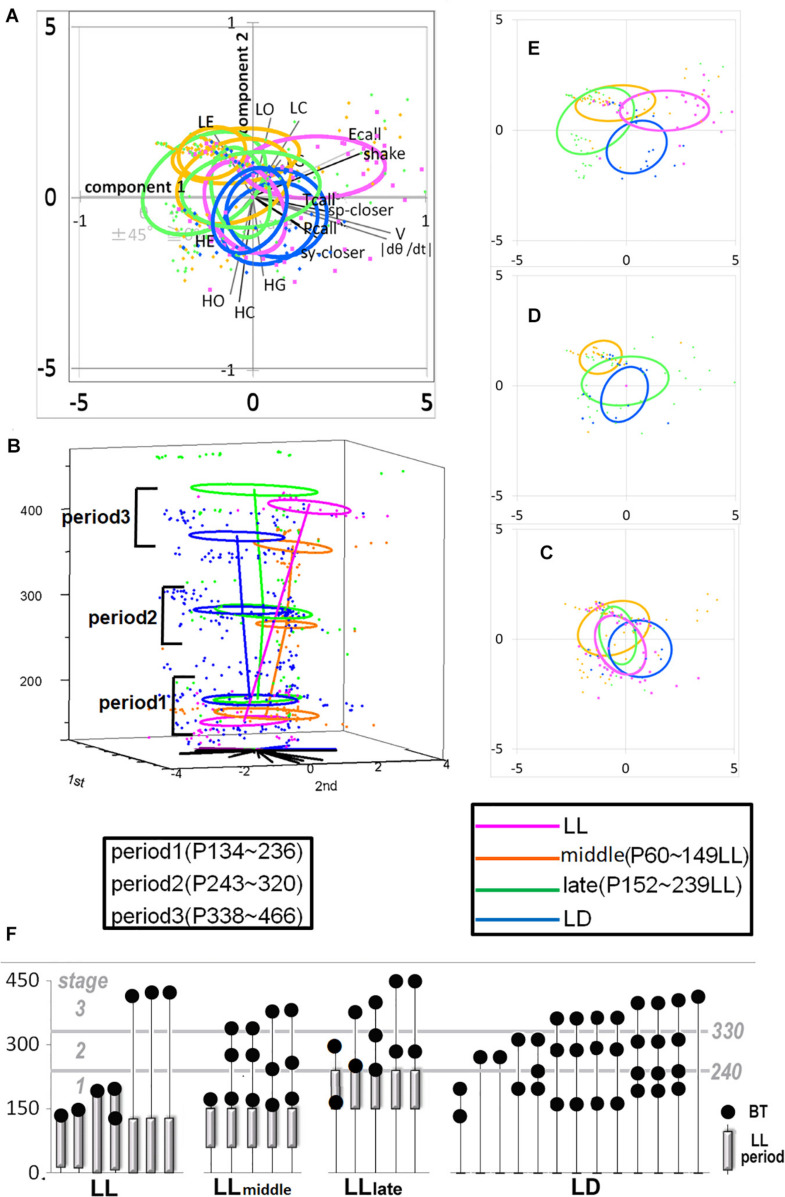
The developmental difference of four groups, LL. Middle, Late, and LD. Developmental linear regression in the 3D (PCA 1st–2nd-age) space. **(A)** All the top view components in the 1st and 2nd coordinates. **(B)** All the side view in 3D space. **(C–E)** Each group distribution summarized using an ellipse regression in period 1 **(C)**, 2 **(D)**, and 3 **(E)**. **(F)** The photic cyclic regulation of each subject under LD, Early, Late, and LD condition and the days of behavior test were indicated by a closed circle. The photic conditions until the day of behavior test were indicated by a bar. The period under LD condition was without a bar.

#### Video Analysis

Five cameras were used for video recording. Video recorded data was converted to JPEG images and WAVE sound files using TMPGEnc (Pegasys, Japan) and then transferred into ImageJ (NIH, United States) files for every second of the trials. The head center and forehead (in most cases, nose-head) position (x, y, and z coordinates) was plotted and data were saved into Excel (Microsoft, United States) files. The line connecting two points—the subject’s head center and an arbitrary forehead part (mostly nose-head)—was used to signify the subject’s view direction (phi) and determine their head rotational speed [absolute [d(phi)/dt]]. Local preference (LP) per space of eight defined parts of the test cage as shown in LE, LC, LG, LO, HE, HC, HG, HO of Figure 1B.

**FIGURE 3:**
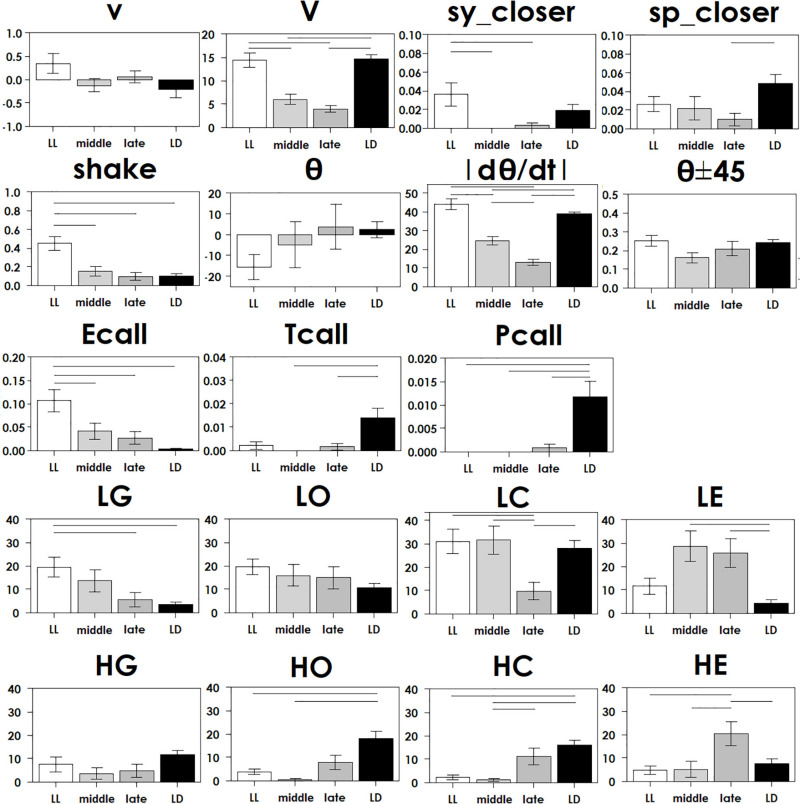
Each parameter in Table 2 was compared in four groups with ANOVA following Tukey’s test at each stage, with statistical significance (*p* < 0.05) indicated by a bar.

Vocal spectrogram visualized by Syrinx (kindly provided by Dr. John Burt from the University of Washington, United States) was used to define 17 call types. The calls were divided into three categories: Pcall, purportedly social antiphonal affinity expression but including “anxious-like” meaning (phee only) (Pistorio et al., 2006; Yamaguchi et al., 2010; Koshiba et al., 2011, 2013c; Senoo et al., 2011); Ecall, seemingly “strained” (egg, highegg, bass, high, higheggbass, or strong alert call “gugaga”) (Pistorio et al., 2006; Yamaguchi et al., 2010; Senoo et al., 2011; Koshiba et al., 2013c) and Tcall, supposedly “feeling affinity” (trill, peep, short, short-combination, trillphee, twitter, twitterhead, U, tsik, tsikstring, cat, hana) (Cross and Rogers, 2006; Pistorio et al., 2006; Senoo et al., 2011; Koshiba et al., 2013c) (Figure 1C). All the defined parameters are listed in Table 2 and were integrated in PCAby correlation matrix (Excel) as the 1st PCA. The longitudinal behavioral development was represented in 3D space (X, Y: the 1st and 2nd component of the 1st PCA, Z: day-age) using software Origin 7.5 (Origin Pro, United States). The develop-mental trajectory estimation was lined between the data set averages per age stage. We showed a variance approximation ellipse whose center is the average of the PCA score plots per group per age stage and whose long or short axis equals the factor loading vectors extended from the average after the second 3D-PCA for the 1st PCA scores by variance–covariance matrix. To determine the approximate contribution of each parameter for the most representative scores of the 1st PCA on the 1st and 2nd components projection plane (contracted with 24% contribution), the factor loading vector was visualized only plus direction with minus vector omitted, then multiplied by our setting maximum value of graph (Figure 3A) to be compared with the projected regression lines.

**TABLE 2:** Parameters for principal component analysis.

Abbreviation	Content of parameter for PCA
Shake	Shaking (alert) behavior frequency
V	Head moving velocity
V	Velocity to other
θ	Head horizontal angle to other
| dθ/dt |	Rotation velocity of θ
θ ± 45°	Frequency of heading to another
LE	Lower preference (Escape space)
LC	Lower preference (Center space)
LG	Lower preference (Grouped space)
LO	Lower preference (Other space)
HE	Higher preference (Escape space)
HC	Higher preference (Center space)
HG	Higher preference (Grouped space)
HO	Higher preference (Other space)
sy-closer	Synchronized approach-to-other frequency
sp-closer	Spontaneous approach-to-other frequency
Ecall	Ecall (alert) frequency
Tcall	Tcall (social affinity) frequency
Pcall	Anxiety help call frequency

## Results

To understand how constant light experience (LL) during specific periods of marmoset’s early development, we applied PCA with correlation matrix of four conditions and compared the resultant variance ellipses based on PCA with variance-co-variance matrix and eigenvectors multiplied by eigenvalues set as the ellipse major and minor axes in each developmental stage periods (I, II, and III, given in Table 1). This is summarized in Figures 2A–E. For the longitudinal follow up of individual animal development, we combined the PCA plots from three age-stages into a 3D time-space with vertical axis set as age. This visualization compared age-specific behaviors revealing that compared to LD (regarded as normal control) or LL (another extreme) which started almost after born and the longest experience), the Middle group development leaned closer to LL and the Late group development leaned closer to LD. According to the factor loading vector directions, the beginning age stage showed all the groups expressed around the average center (Figure 2C), then the final age stage showed LL to alert (Ecall, Shake) and body and head rotation to other active (V, | d(theta)/dt|) (Figure 2E), LD to local preference higher (HO, HC, HG). Three groups, LL, Middle and Late, with a certain LL experience present, leaned to local preference lower direction (LE, LO, LC) as clustering but lower loading factors. The LL characterized-like factor, alert profile (Senoo et al., 2011; Koshiba et al., 2015) seemed descended, simultaneously with activity reduction in Late group than Middle group. The significance of all the combination were described with provability of Wilk’s lambda analysis in Table 3. Each parameter value was compared in the period 3 of groups, LL, Middle, Late and LD by one-way ANOVA with *post hoc* Tukey HSD. Significant pairs of the comparison are shown in Figure 3 [Shake: *F*(3, 180) = 10.663, *P* < 0.001, V: *F*(3, 180) = 30.919, *P* < 0.001, v: *F*(3, 180) = 1.4703, *P* = 0.2242, θ: *F*(3, 180) = 0.9423, *P* = 0.4214, | dθ/dt | : *F*(3, 180) = 65.231, *P* < 0.001, θ ± 45°: *F*(3, 180) = 1.7223, *P* = 0.164, LE: *F*(3, 180) = 7.8175, *P* < 0.001, LC: *F*(3, 180) = 5.6913, *P* < 0.001, LG: *F*(3, 180) = 5.5056, *P* < 0.01, LO: *F*(3, 180) = 1.0881, *P* = 0.3555, HE: *F*(3, 180) = 4.3802, *P* < 0.01, HC: *F*(3, 180) = 5.6913, *P* < 0.001, HG: *F*(3, 180) = 2.4255, *P* = 0.06718, HO: *F*(3, 180) = 8.1169, *P* < 0.001, Ecall: *F*(3, 180) = 10.663, *P* < 0.001, Tcall: *F*(3, 180) = 4.8312, *P* < 0.01, Pcall: *F*(3, 180) = 5.934, *P* < 0.001, sy_closer: *F*(3, 180) = 4.5081, *P* < 0.01, sp_closer: *F*(3, 180) = 3.4726, *P* < 0.05). Generally, the alert behaviors (Ecall, Shake) were significantly frequent in LL and less in Middle, Late and LD. Pcall was clearly frequent in LD but not any other groups. Meanwhile, higher positioning, HO and HC were significantly more frequent in LD than Middle and LL.

**TABLE 3:** The probabilities of Wilks’ lambda analysis.

Comparison		Period 1	Period 2	Period 3
Early	Late	0.5242	1.25E-05***	0.0063**
Early	LD	5.08E-10***	9.09E-14***	2.20E-16***
Early	LL	0.009526**	–	2.47E-05***
Late	LL	0.5124	–	8.22E-09***
LD	LL	0.001027**	–	2.85E-09***
LD	Late	0.003768**	0.03273*	3.39E-15***

*p < 0.05, **p < 0.01, and ***p < 0.001.

## Discussion

We explored the effects of photic environmental rhythm loss among non-human primates and analyzed its effect on their social development by utilizing methods involving social affective states and visualization based on digitized behavior under a particular social context, BOUQUET (Senoo et al., 2011; Koshiba et al., 2013a, b, c, d, 2015; Karino et al., 2020; Tao et al., 2020). To focus on the major impacts of developmental disorders in socialization, we identified a set of social behavior factors for analysis involving the social view (theta), the rotation velocity, [d(theta)/dt|], approach (sy-closer, sp-closer), vocalization translated with three types of emotion (Ecall, Pcall, Tcall), and typical alert body motion (Shake). LL marmosets elicited a set of alert behaviors, egg-like calls (Ecall) and Shake in adulthood, responding patterns in unfamiliar social contexts, which were similarly reported in previous and more recent studies. The previous report and review implied a susceptible period present in the ages 3–5 months old in post weaning juvenile stage about the photic environmental rhythm loss (Senoo et al., 2011; Koshiba et al., 2013c, 2015). These patterns suggested the semantics of psychological states of subjects and qualitatively assessed it, possibly interpreted as a stress-related behaviors revealing subtle differences in LL from LD groups as increased alert and hyperactivity at the social context in adolescence.

In a similar study of other animals, adult rats regulated with chronic constant light exhibited depressive and anxiety behavior as grooming in the open-field test and an anhedonia-like expression in a sucrose consumption test and with decreasing cellular activation visualized by c-Fos immunohistochemistry in the biological clock nerve center, the suprachiasmatic nucleus (SCN) (Tapia-Osorio et al., 2013). Grooming in rodents was reported about its complexity as a possible neuropsychological behavior marker. It was suggested in some aspects that increasing and decreasing of self-grooming could be translated as anxiety- and depression-like states, respectively (Denmark et al., 2010; Kalueff et al., 2016). The primates experienced LL might visualize in Figures 2, 3 consistently exhibited comparative depressive- or anxiety-like expression, not like the rodents grooming but in localizing preference of lower areas (Senoo et al., 2011), possibly translated motor suppressed function without the species specific climbing exploration under the gravity-dependent potential energy though it is merely our hypothesis. Comparing to LD group, the three groups experienced LL during development significantly expressed less Pcall which was reported an anxiety-like and social contact behavior (Cagni et al., 2012; Figure 3). Consequently, the impact of constant light during development might express certain socio-emotional aberrance.

There were four conditional groups, LL (P0-around 150), Middle [LL(P60–149)] as a peri-weaning and early juvenile period, Late [LL(P150–239)] as a late juvenile to early adolescent period and LD (no LL). A certain conditional limitation among the infants in our colony led to imbalances in the sample number, sex, test number and age affecting the procedures of constant light experiences and social tests as shown in Table 1. Some sexual differences were observed in the current sample which leaned toward males in Middle and Late might be considered as a result distribution. It has been known that females have higher risks of anxiety and depressive disorders during adolescence and early adulthood, caused by brain dimorphisms and hormonal influences (Altemus et al., 2014). Both Middle and Late groups with males were more inclined to moving-less [opposite direction of V and | d(theta)/dt|] than LD and LL shown in Figure 2. Consequently, the LL characterized-like factor, supposedly “alert” behavior, represented by higher frequency of Ecall and Shake suggested in a previous report could be repeatedly confirmed in this study with further graduating distribution from LL as the strongest, Middle and Late reduced in turn during the final adult age stage in Figures 2, 3. The “susceptible period of photic rhythm loss experience” was implied as the previously hypothetic period—postnatal 3–5 months old—but also the earlier ages included. Furthermore, the different expressions observed in the Late group might suggest that there might be several different mechanisms of susceptible period learning when the subjects were exposed to constant light.

The common profile of three groups with LL experiences against LD was localizing preference in higher position of the test cage (HC, HO, HG) not away from the other (shorter HE). In the single parameter comparison analysis, HO and HC were significantly frequent in LD but less often in Late, Middle and LL (Figure 3). These findings could be successfully supported in LL characterized alert behavior with Ecall and Shake as shown in our BOUQUET visualization and the reduction of higher local preference, whose meanings might be assumed as “depressive” with potential energy restriction. Animal model studies of depression have contributed to reveal pathogenesis and the development of multidirectional treatment strategies (Demin et al., 2019). Traumatic life experiences induces dysregulation in the hypothalamic-pituitary-adrenal (HPA) axis with cortisol and the locus caeruleus/norepinephrine-sympathetic nervous system (LC/NE-SNS) when the immune system activates (Pervanidou and Chrousos, 2012). These are the reasons why this current study is significant to understanding and supporting the well-being of humans in their early stages of development.

Developmental disorders have been considered contributory to locomotion development abnormalities (Teitelbaum et al., 2004; McCleery et al., 2013; Koshiba et al., 2016). The central nervous systems of these functions elicited in social context after several timings of LL experiences apparently involved the limbic system including the brainstem, the cerebellum, the midbrain, the interbrain and the forebrain (Hafizi et al., 2019) and genetic factors (Finucane and Myers, 2016). Considering the interventions through comprehensive treatment approaches, digital visualization of each individual’s specific developmental trajectory may be supported by adequate treatment design (Hickie et al., 2019). In this trial, we used 19 types of social behavioral factors, understanding that we would have less significant findings if we used a smaller number or different factor sets (data not shown). The reason of this factor might suggest that there the outcomes caused by early photic rhythm loss experiences on social behavior functions which were reported in the Japanese survey of children situation as one of the most sleep-deprived societies (Kohyama, 2014) are significant. Our study adds to other significant research such as the one involving adolescents at clinical high-risk of psychosis symptoms, psychosocial functioning and the longitudinal course of illness (Lunsford-Avery et al., 2017), and another which proposed a strong relation between sleep problems and social developmental disorders, attention-deficit hyperactivity disorder (ADHD) (Um et al., 2017).

The previous and current study chose a disciplined strategy of individually housed subject observation to simply focus on photic environmental influences and to avoid the most disturbing risk of diversified family effects. The human surrogate mother took rich and similar attachment over different infants by repeatedly warm handling with feeding and leading their feces and urine cares multiple times per day till weaning period. As the reason of this strategy, our other study with the similar socio-contextual behavior analysis revealed complex social influences to juvenile marmosets by both parents and siblings interactively (Koshiba et al., 2013c).

Another report of ours visualized social learning memory effects by the similar behavioral tests in adult common marmosets who had met each other previously (Koshiba et al., 2011). Consequently, we controlled the social experience within a few times in the current study to reduce the social memory effect even though our colony had a subject condition which cannot control the meeting repetition time equally. Considering the importance of witness feedback in our social behavior modification (Rechdan et al., 2018), the current subject number made it difficult to analyze the intervention statistically, so we attempted to counterbalance the variable photic conditions in the same meeting experiment.

As reported in a previous study (Moriceau and Sullivan, 2004, 2005) involving a rat’s susceptible learning model, different mechanisms exist as neonatal rapidly learning the odor-based maternal attachment by hyper-functioning noradrenergic locus coeruleus during sensitive period, or fear-conditioning and passive avoidance in learning odor aversions during post sensitive period by amygdala development. Such as age-dependent biological mechanisms could be hypothesized as a certain reason of differences in our experimental groups, LL, Middle, Late, and LD.

Sexual dimorphism describes developmental influence different in circadian behavior and physiology (Anderson and FitzGerald, 2020). Female mice amplitude of behavioral oscillation was reported greater than male one (Royston et al., 2014). The variation contributors between sexes are thought not only hormones and the receptors in the suprachiasmatic nucleus or the circadian network but also the gut microbiota (Liang et al., 2015). Our study had a limitation in subjects’ sexual bias, which might affect each group distribution.

Longer exposure to photic cycles among humans revealed crucial influences in the food intake and weight development of infants who were admitted in a neonatal intensive care unit (NICU) (Vásquez-Ruiz et al., 2014). Our challenge is to development a holistic measurement system for preterm infants in the NICU by visually analyzing the complexities involving vocal behavior variation, blood oxygen saturation percentage and pulse rate modulation (Tao et al., 2019). Similar to our study but focusing more to involve human care with holistic intervention, diet, education and supportive medicine, there is need to further develop some automatic behavioral measurement systems using image, sound or human motion sensors (Melo et al., 2015).

## Conclusion

We explored susceptible periods of constant ambient light among the LL common marmoset group during their early development to understand how the environment affected their socialization. An examination of the socio-developmental behaviors of the subjects was carried out using BOUQET based on Principal Component Analysis. Group LL (P0-around 150d) expressed highly “typical” alert behavior, then, Middle (P60–149d), and Late (P150–239d) groups which had gradually reduced alert behaviors. We observed that the subjects commonly suppressed higher positioning behaviors over three groups against LD (no LL experience). This limited study might suggest differently varied mechanisms of LL susceptible learning systems depending on their developmental stages.

## Data Availability Statement

The datasets generated for this study are available on request to the corresponding author.

## Ethics Statement

The animal study was reviewed and approved by the Animal Care and Use Committee of the Tokyo Metropolitan Institute for Neuroscience. Written informed consent was obtained from the owners for the participation of their animals in this study.

## Author Contributions

MK generally did all the work. AW-S, GK, and SO conducted the behavior examination and analyses. YH, IT, TK, and SU experimentally reared animals following the procedures of the experiment. All members designed the treatments. HT reared the animals and supervised their treatments with YK’s clinical and comparative supervision. All authors contributed to the article and approved the submitted version.

## Conflict of Interest

The authors declare that the research was conducted in the absence of any commercial or financial relationships that could be construed as a potential conflict of interest.
